# Supplementary material for: Hot spot prediction in protein-protein interactions by an ensemble system
Source: BMC Syst Biol. 2018 Dec 31;12(Suppl 9):132. doi: 10.1186/s12918-018-0665-8 (PMC6311905; doi:10.1186/s12918-018-0665-8)
Supplement: Supplementary file 2 — All relevant MATLAB code. A simple Matlab implement of our predictor. (ZIP 19,742 kb) [file 12918_2018_665_MOESM2_ESM.zip › Additional file 2/Read ASA/ASA website/SKEMPI/2SICI.htm]

 NetSurfP - Protein Surface Accessibility and Secondary Structure Predictions

  

|  |  |  |
| --- | --- | --- |
|  |  | NetSurfP - Protein Surface Accessibility and Secondary Structure Predictions  Technical University of Denmark |

  


---

```
# For publication of results, please cite:
# A generic method for assignment of reliability scores applied to solvent accessibility predictions.
# Bent Petersen, Thomas Nordahl Petersen, Pernille Andersen, Morten Nielsen and Claus Lundegaard
# BMC Structural Biology 2009, 9:51 doi:10.1186/1472-6807-9-51
#
# Column 1: Class assignment - B for buried or E for Exposed - Threshold: 25% exposure, but not based on RSA
# Column 2: Amino acid
# Column 3: Sequence name
# Column 4: Amino acid number
# Column 5: Relative Surface Accessibility - RSA
# Column 6: Absolute Surface Accessibility
# Column 7: Z-fit score for RSA prediction
# Column 8: Probability for Alpha-Helix
# Column 9: Probability for Beta-strand
# Column 10: Probability for Coil
E Y  2SICI                  1    0.731 156.236  -1.541   0.003   0.003   0.994
E A  2SICI                  2    0.451  49.667  -1.052   0.005   0.045   0.951
E P  2SICI                  3    0.350  49.608  -0.999   0.018   0.088   0.893
B S  2SICI                  4    0.268  31.351  -0.941   0.021   0.279   0.699
B A  2SICI                  5    0.136  14.943  -0.508   0.004   0.616   0.381
B L  2SICI                  6    0.071  12.963   0.590   0.011   0.918   0.071
B V  2SICI                  7    0.109  16.723   0.719   0.001   0.959   0.040
B L  2SICI                  8    0.038   6.866   0.674   0.001   0.959   0.040
B T  2SICI                  9    0.079  10.943  -0.026   0.001   0.900   0.099
B V  2SICI                 10    0.070  10.759  -0.146   0.003   0.718   0.279
B G  2SICI                 11    0.129  10.192  -0.145   0.022   0.552   0.426
E K  2SICI                 12    0.425  87.464   0.219   0.021   0.279   0.699
E G  2SICI                 13    0.351  27.600  -1.171   0.018   0.088   0.893
E V  2SICI                 14    0.554  85.211  -0.998   0.018   0.047   0.935
E S  2SICI                 15    0.528  61.846  -1.205   0.018   0.047   0.935
E A  2SICI                 16    0.382  42.129  -1.394   0.053   0.043   0.903
E T  2SICI                 17    0.555  77.020  -1.197   0.053   0.043   0.903
E T  2SICI                 18    0.427  59.183  -0.874   0.053   0.043   0.903
E A  2SICI                 19    0.429  47.331  -1.233   0.113   0.043   0.844
E A  2SICI                 20    0.372  41.027  -0.397   0.113   0.087   0.800
E P  2SICI                 21    0.312  44.301  -0.260   0.064   0.216   0.721
B E  2SICI                 22    0.305  53.353  -0.278   0.021   0.451   0.528
B R  2SICI                 23    0.272  62.242   0.258   0.023   0.655   0.322
B A  2SICI                 24    0.213  23.495  -0.101   0.018   0.846   0.136
B V  2SICI                 25    0.096  14.786   0.264   0.011   0.918   0.071
B T  2SICI                 26    0.213  29.599   0.261   0.011   0.918   0.071
B L  2SICI                 27    0.044   7.965   0.591   0.011   0.918   0.071
B T  2SICI                 28    0.239  33.149   0.290   0.018   0.846   0.136
B C  2SICI                 29    0.038   5.279  -0.978   0.003   0.718   0.279
E A  2SICI                 30    0.349  38.471   0.569   0.005   0.336   0.660
B P  2SICI                 31    0.232  32.892  -0.689   0.004   0.138   0.858
E G  2SICI                 32    0.441  34.722  -0.065   0.018   0.047   0.935
E P  2SICI                 33    0.565  80.117  -2.217   0.018   0.019   0.964
E S  2SICI                 34    0.607  71.164  -2.187   0.018   0.019   0.964
B G  2SICI                 35    0.256  20.147  -2.025   0.018   0.019   0.964
E T  2SICI                 36    0.432  59.877  -1.012   0.018   0.019   0.964
B H  2SICI                 37    0.195  35.507   0.266   0.018   0.019   0.964
E P  2SICI                 38    0.463  65.671  -0.620   0.109   0.005   0.886
E A  2SICI                 39    0.394  43.474   0.355   0.053   0.005   0.942
B A  2SICI                 40    0.114  12.552  -0.105   0.600   0.003   0.397
E G  2SICI                 41    0.483  38.004   0.217   0.782   0.003   0.216
E S  2SICI                 42    0.512  59.971   0.843   0.858   0.002   0.139
B A  2SICI                 43    0.062   6.854   0.216   0.923   0.002   0.076
B C  2SICI                 44    0.030   4.282   0.219   0.970   0.001   0.030
E A  2SICI                 45    0.463  51.023   0.812   0.970   0.001   0.030
E D  2SICI                 46    0.398  57.280   1.231   0.923   0.002   0.076
B L  2SICI                 47    0.053   9.649   1.144   0.923   0.002   0.076
E A  2SICI                 48    0.462  50.879   0.908   0.858   0.002   0.139
E A  2SICI                 49    0.598  65.911  -0.198   0.694   0.003   0.303
B V  2SICI                 50    0.160  24.638  -0.334   0.339   0.016   0.645
E G  2SICI                 51    0.564  44.426  -1.517   0.115   0.016   0.868
B G  2SICI                 52    0.264  20.769  -1.866   0.018   0.019   0.964
E D  2SICI                 53    0.463  66.776  -0.002   0.018   0.047   0.935
B L  2SICI                 54    0.115  21.002  -0.332   0.052   0.084   0.864
E N  2SICI                 55    0.701 102.597   0.253   0.052   0.084   0.864
E A  2SICI                 56    0.470  51.761   0.297   0.052   0.084   0.864
B L  2SICI                 57    0.061  11.261  -0.045   0.053   0.043   0.903
E T  2SICI                 58    0.398  55.147   1.026   0.113   0.043   0.844
E R  2SICI                 59    0.546 125.080   0.003   0.053   0.043   0.903
E G  2SICI                 60    0.592  46.559  -0.201   0.018   0.019   0.964
E E  2SICI                 61    0.678 118.516  -0.285   0.016   0.005   0.979
E D  2SICI                 62    0.706 101.792  -0.926   0.005   0.015   0.979
E V  2SICI                 63    0.286  43.927  -0.498   0.018   0.047   0.935
B M  2SICI                 64    0.230  46.003  -0.039   0.019   0.141   0.840
B C  2SICI                 65    0.055   7.694   0.665   0.019   0.141   0.840
E P  2SICI                 66    0.283  40.200   0.138   0.021   0.279   0.699
B M  2SICI                 67    0.282  56.468   0.274   0.021   0.451   0.528
E V  2SICI                 68    0.374  57.422  -0.188   0.022   0.552   0.426
B Y  2SICI                 69    0.187  39.855  -0.013   0.021   0.451   0.528
E D  2SICI                 70    0.488  70.335  -0.015   0.004   0.197   0.799
B P  2SICI                 71    0.280  39.718  -0.654   0.005   0.336   0.660
B V  2SICI                 72    0.070  10.820  -0.018   0.002   0.816   0.182
E L  2SICI                 73    0.254  46.507   1.014   0.001   0.959   0.040
B L  2SICI                 74    0.066  12.085   0.412   0.001   0.959   0.040
B T  2SICI                 75    0.229  31.832   0.686   0.001   0.959   0.040
B V  2SICI                 76    0.065   9.975  -0.008   0.001   0.900   0.099
E D  2SICI                 77    0.443  63.851   1.232   0.001   0.900   0.099
B G  2SICI                 78    0.082   6.485  -0.670   0.001   0.900   0.099
E V  2SICI                 79    0.298  45.833   0.515   0.001   0.900   0.099
B W  2SICI                 80    0.117  28.114  -0.959   0.003   0.718   0.279
E Q  2SICI                 81    0.590 105.410   0.191   0.004   0.197   0.799
E G  2SICI                 82    0.547  43.025  -1.855   0.018   0.088   0.893
E K  2SICI                 83    0.482  99.065   0.522   0.004   0.514   0.481
E R  2SICI                 84    0.491 112.439   0.209   0.003   0.718   0.279
B V  2SICI                 85    0.168  25.822   0.897   0.021   0.756   0.223
E S  2SICI                 86    0.438  51.298   0.492   0.021   0.756   0.223
B Y  2SICI                 87    0.270  57.656   0.223   0.023   0.655   0.322
E E  2SICI                 88    0.579 101.134   0.597   0.079   0.592   0.329
E R  2SICI                 89    0.336  76.898   0.844   0.079   0.592   0.329
E V  2SICI                 90    0.421  64.708  -0.172   0.079   0.592   0.329
B F  2SICI                 91    0.239  48.068  -0.064   0.069   0.386   0.545
B S  2SICI                 92    0.248  29.101  -1.339   0.125   0.227   0.648
E N  2SICI                 93    0.390  57.111  -0.828   0.118   0.150   0.732
E E  2SICI                 94    0.410  71.592   0.191   0.135   0.317   0.548
B C  2SICI                 95    0.101  14.166  -0.782   0.074   0.484   0.442
B E  2SICI                 96    0.286  49.912  -0.168   0.087   0.683   0.230
B M  2SICI                 97    0.236  47.264  -0.800   0.087   0.683   0.230
E N  2SICI                 98    0.488  71.370  -0.207   0.022   0.552   0.426
E A  2SICI                 99    0.479  52.742  -1.087   0.021   0.279   0.699
E H  2SICI                100    0.565 102.737  -1.121   0.020   0.205   0.775
E G  2SICI                101    0.476  37.501  -0.793   0.019   0.141   0.840
E S  2SICI                102    0.424  49.740  -1.266   0.019   0.141   0.840
B S  2SICI                103    0.272  31.843  -0.349   0.022   0.552   0.426
B V  2SICI                104    0.216  33.199  -1.309   0.023   0.655   0.322
B F  2SICI                105    0.210  42.167  -0.155   0.023   0.655   0.322
E A  2SICI                106    0.513  56.500  -0.694   0.022   0.552   0.426
E F  2SICI                107    0.449  90.054  -1.913   0.005   0.015   0.979

Explain the output.  Go back.
```

---
